# A dreaded delight: A longitudinal qualitative interview study of paternal childbirth fear during the transition to fatherhood

**DOI:** 10.18332/ejm/142783

**Published:** 2021-11-10

**Authors:** Margareta Johansson, Michael B. Wells, Li Thies-Lagergren

**Affiliations:** 1 Department of Women’s and Children’s Health, Uppsala University Hospital, Uppsala University, Uppsala, Sweden; 2 Department of Women’s and Children’s Health, Karolinska Institutet, Stockholm, Sweden; 3 Department of Midwifery Research - Reproductive, Perinatal and Sexual Health, Lund University, Lund, Sweden; 4 Department of Obstetrics and Gynecology, Helsingborgs Lasarett, Helsingborg, Sweden

**Keywords:** coping strategies, interviews, longitudinal, fathers, childbirth fear, reasons for fear

## Abstract

**INTRODUCTION:**

Childbirth is a life-changing event where fathers desire being involved. As fathers take a more active role, fear of childbirth can arise. The aim of this study was to explore fathers’ experiences of childbirth fear during pregnancy and after the birth of their baby.

**METHODS:**

This was a qualitative longitudinal prospective study that included 14 interviews with seven fathers, one during the prenatal period, and one follow-up interview after childbirth. Data were analyzed using thematic analysis according to Braun and Clark.

**RESULTS:**

The main theme ‘Being vulnerable during the transition to fatherhood’ was based on the perception of childbirth as risky with threats toward the woman’s and baby’s health, not being able to give and receive enough support, unable to handle the unknown process of birth, and not being a good father. Helpful strategies for coping with fear were to talk about fear, to learn more about childbirth and techniques on how to handle fear, and to avoid dealing with fear. Fathers’ fear of childbirth changed after the birth of their baby. Their thoughts of another childbirth did not evoke the same strong feelings of fear. Issues important for the reduction of childbirth fear were: receiving professional support, actively taking part in the childbirth process, and the partner having an uncomplicated birth.

**CONCLUSIONS:**

Fathers with childbirth fear regarded childbirth as risky, but they expressed helpful coping strategies. After the birth of their baby, they became less fearful. Quality of fear-reducing support to expectant fathers may influence how they cope with their transition into fatherhood.

## INTRODUCTION

The World Health Organization promotes increased involvement of fathers in maternal and child healthcare^1^. As fathers take a more active role during birth, feelings of anxiety and fear of childbirth (FOB) can arise^2,3^. High levels of fear are likely to have an impact on expectant fathers’ own emotional health. When fathers experience FOB, it may also influence their ability to be emotionally and physically supportive and ‘available’ to their partner^2^. Studies have described that 13% of fathers suffer from pathological FOB^4,5^. Prevalence rates may differ due to several reasons including diverse cultural contexts^3,6,7^, using different screening instruments^7^, different maternity care organizations^6^, and different definitions of childbirth fear^8^. Associated factors for fathers with FOB include expecting their first child^4,9^, preference for a caesarean delivery^9,10^, experience of a traumatic birth^11^ such as emergency caesarean delivery^10^, and not being born in the country they currently live in^4^. High levels of fear are likely to have an impact on expectant fathers’ own emotional health and their experience of childbirth. When expectant fathers experience FOB, it may also negatively influence their ability to be emotionally and physically supportive and ‘available’ to their partner^2,11,12^. Previous research suggests that the quality of fear-reducing support to expectant fathers may influence how they cope with their transition into fatherhood^13^. Thus, it is vital to understand in what ways their health is affected and for how long FOB persists, to be able to provide adequate support to fathers expressing FOB. Longitudinal qualitative research studying expectant fathers’ experiences of FOB is lacking. Therefore, the aim of the study was to explore fathers’ experiences of childbirth fear during pregnancy and after the birth of their baby.

## METHODS

### Study design

A qualitative longitudinal prospective study design was employed to explore how fathers interpret and respond to their fear of childbirth over time, as well as identify and understand the meaning of the change into fatherhood^14,15^. Therefore, this study included an interview during the prenatal period and a follow-up interview 6-13 weeks after childbirth (Table 1).

**Table 1:** Background information

*Characteristics*	*Study participant number*						
	*1*	*2*	*3*	*4*	*5*	*6*	*7*
Paternal age (years)	26	32	29	45	29	33	34
Number of previous children of expectant fathers	1	0	0	1	0	1	0
Number of previous children of partner	3	0	0	1	0	1	0
Gestational week at the time of first interview	31+2	36+5	32+3	38+1	34+2	17+4	32+5
Gestational week at birth of their current baby	42+1	39+4	42+2	39+2	39+4	36+1	38+6
Number of weeks after birth for the follow-up interview	6	11	8	13	12	11	9

### Setting and participants

The study participants were recruited between 2014 and 2017 through five antenatal care units in Stockholm, Sweden. A poster with an invitation to participate in the study was placed in each unit’s waiting room. Expectant fathers with self-reported FOB and with sufficient knowledge of Swedish or English to conduct an interview, were included. Expectant fathers who met the inclusion criteria and were interested in participating, contacted the first author by telephone or e-mail and arranged a time for the interview session. In total, seven expectant fathers participated in the study. Swedish was used in all interviews but one, where English was used.

### Measures and variables

The audio-recorded telephone interviews were conducted at two time points and resulted in 14 interviews. The interviews at Time 1 (T1) were conducted between October 2014 and February 2017 during pregnancy. A follow-up interview at Time 2 (T2) was conducted after the birth of their baby between February 2015 and September 2017 (Table 1). The interviews, which followed an interview guide (Table 2), lasted on average 57 minutes during T1, and 48 minutes during T2.

**Table 2:** Questions in the interview guide

*Time-point*	*Main and follow-up questions*
**During pregnancy**	How would you describe your experience of childbirth fear?
	Follow-up questions
	Which reasons are behind your fear of childbirth?
	Which issues increase your fear of childbirth?
	Which issues decrease your fear of childbirth?
	What types of professional support do you desire and/or receive?
**After childbirth**	How would you describe your birth experience?
	Follow-up questions
	What impact did your fear have on you during childbirth?
	Did the aspects of your fear for childbirth become true?
	If you prepared yourself for your child’s birth, what was relevant for you?

During the first interview (T1), participants provided details regarding: age, civil status, having a planned pregnancy, single or multiple pregnancy, due date, and number of previous children. In addition, they assessed their current feelings regarding their childbirth fear using the Fear of Birth Scale (FOBS)^7^, during pregnancy (T1) and 6-13 weeks after the birth of their baby. By responding to the question ‘How do you feel right now about the upcoming birth?’, the study participants described their experience of: 1) fear, and 2) worry. By combining the two anchors, *no fear* (0 mm)-strong *fear* (100 mm) and *calm* (0 *mm)-worried* (100 mm), a mean score was estimated^7^. The cut-off point of 60 mm or higher has been found to be suitable for identifying FOB^16^, and FOBS has previously been used in investigations of fathers’ FOB experiences^4^. At T2, the fathers re-completed the childbirth fear assessment according to FOBS for a potential future birth of another child, as well as provided their current baby’s day of birthday.

### Statistical analysis

The qualitative longitudinal prospective data were analyzed using thematic analysis according to Braun and Clarke^17^, as it is a flexible method appropriate to offer rich and compelling insights into experiences within health and wellbeing research^18^. Thematic analysis contains six phases: 1) the authors familiarized themselves with the data, which included transcribing data verbatim by the first author, reading and re-reading the data, and noting initial ideas together with the middle author; 2) generating initial codes with relevant features according to the aim and collating data relevant to each code; 3) codes were sorted into potential themes (these were, for T1, personal attributes, items related to FOB, supportive aspects, impact of partner and parenthood, and for T2, reasons for FOB, strategies for coping with FOB, desired and received professional support and childbirth experience); 4) potential themes were reviewed by all authors, triangulated and collapsed from the two designated time-points into the new themes and were described via subthemes; 5) the main theme ‘Being vulnerable in the transition into fatherhood’ was explored, which described the fathers’ FOB during the partner’s pregnancy and after the birth of their baby (Table 3); and 6) include a selection of vivid, compelling quotes in the findings of the study^17^.

**Table 3:** Description of items that contextualized the main theme

*Themes*	*Items*
**Overarching theme**	Being vulnerable during the transition into fatherhood.
**Themes**	The risky childbirth as a source of fear.
	Helpful strategies for coping with fear.
**Subthemes**	Threats towards the woman’s and the baby’s health and wellbeing.
	To talk about the fear.
	Not being able to give the partner sufficient support.
	To learn more about childbirth.
	Not receiving sufficient professional support.
	To learn techniques how to handle the fear.
	Not being able to handle the unknown process of childbirth.
	To avoid dealing with the fear.
	Not knowing how to become a good father.

The study was approved by the chief manager of the antenatal care units, and by the Ethical Review Board in Stockholm. All participants were given written and oral study information, and informed that their participation was voluntary, that they were free to withdraw from participating without any explanation, and if any more questions arose, they were informed to make contact whenever they wanted. Data were processed with confidentiality, and anonymity was protected. Data is securely stored on university servers according to standard university data storage protocols.

## RESULTS

The participants were aged 26-45 years (mean: 32.6 years), all cohabiting with their partner, who all had a planned, simplex pregnancy. Four of the seven fathers were expecting their first baby (Table 1). All babies were born spontaneously except for one that was born via vacuum extraction. Three of the births were complicated by preeclampsia, anal sphincter rupture and induction of labor related to pregnancy being overdue.

At T1, expectant fathers had a score of 50-100 mm (mean: 68 mm) on FOBS. When asked about their childbirth fear for having another child in the future, fathers at T2 had a score of 0-57.5 mm (mean: 28 mm) on FOBS (Table 4).

**Table 4:** Assessment of childbirth fear during pregnancy and after the birth of the baby

*Experiences*	*Study participant number*						
	*1*	*2*	*3*	*4*	*5*	*6*	*7*
**Experience of childbirth fear during pregnancy**							
FOBS (mm)*	100	52.5	50	60	70	70	72.5
Experience of fear (mm)**	100	55	40	60	70	70	85
Experience of worry (mm)**	100	50	60	60	70	70	60
**Experience of childbirth fear postpartum if expecting another child in the future**							
FOBS (mm)*	0	12.5	25	57.5	30	32.5	37.5
Experience of fear (mm)**	0	0	30	50	30	35	35
Experience of worry (mm)**	0	25	20	65	30	30	40

* FOBS: fear of birth scale (based on a combination of fear and worry).

** Measured on a 100 mm scale (0 mm = no fear/calm; 100 mm = strong fear/worried).

### Overarching theme: Being vulnerable during the transition to fatherhood

This longitudinal study resulted in the overarching theme ‘Being vulnerable during the transition to fatherhood’ comprising the two themes: 1) ‘The risky childbirth as a source of fear’, and 2) ‘Helpful strategies for coping with fear’ (Table 3).

The overarching theme described participants’ experiences of childbirth fears during pregnancy, labor and birth, and the postnatal period. The concepts of fear and worry were interchangeably used by the participants to describe their childbirth fear. Childbirth fear was considered something real and linked to an external influence, whereas worries were experienced when reflecting on what may arise in relation to childbirth. For example, one participant noted:

*‘You are rarely worried about a tiger, but may be fearful of a tiger.’* (Participant 6)

### Theme 1: The risky childbirth as a source of fear

Fathers were worried about any complications that could result in death or decreased the health of their partner and/ or baby. They feared that their partner would experience childbirth complications, intense labor pain, massive bleeding, and unplanned caesarean delivery. They described how:

*‘… to see everything in a worst-case scenario.’* (Participant 5)

They further associated their fears with a sterile hospital environment, blood sampling, syringes, injections, and surgery. If any of these occurred, fathers imagined they would feel uncomfortable, horrified, leave the room, and/ or faint.

Their baby-related fears included the baby’s health and life, both during labor and after the birth of their baby. If complications with their baby occurred, shock and panic would dominate their birth experience, but they would still be happy for having a baby;

*‘My biggest fear is if my baby comes out dead. What panic! [I] don't know how I would take it. There is a risk that I will leave her and feel badly for not being able to stay by her side.’* (Participant 1)

Fathers stated that these threats were less intense when midwives had supported them by providing sufficient information and explaining the situation adequately. However, stress was experienced when their partner had intense labor pain, was bleeding heavily, and had vaginal ruptures or if the baby needed neonatal resuscitation:

*‘It was a bit stressful when the [midwife] presented something, which looked like a ventouse, but after getting the explanation that it wasn't [a ventouse], but a fetal scalp electrode, I felt calm.’* (Participant 7)

Fathers feared that they might not be able to provide sufficient support to their partner during the upcoming labor and birth, which might lead to feelings of powerlessness and a need for their partners to support them. Instead, the expectant fathers wished to be ‘calm, present, involved in both joy and sorrow, to be able to take responsibility’ and to provide comfort, protection and give sufficient support to their partner during labor and birth. They wanted to be involved in the process of labor through their presence and active involvement with their birthing partner. They wanted to be able to encourage and assist their partner, and after birth, to help each other to take care of their baby. Furthermore, being involved in childbirth was regarded as important in helping fathers feel committed as a parent as well as not disappoint their partner.

During childbirth, fathers were mostly capable of supporting their partner despite their fears. Fathers talked about how they restrained their fears so that their partner would not know how concerned they were about the whole birth process. They reported that they made their partner feel comfortable, assisted with what she needed, motivated her, reminded her of learned techniques, transferring the midwives’ instructions, were positive and present in the room, held her hand and talked in a calm voice:

*‘I was standing next to her holding hands and tried to encourage her. I kept my voice very calm all the time, but I was in total panic. I masked it [the fear] very well because she did not notice anything at the time.’* (Participant 5)

Childbirth fear was also related to midwives not providing sufficient support and attention to the couple. For example, if the midwives did not let the birthing couple come to the hospital when desired, if the hospital was overcrowded, and if they thought they were discharged too early after birth, then fathers felt that they had received insufficient professional birthing support. Childbirth fear was also connected to their fear of not being respected, not receiving clear information, and having a feeling that the birth would result in harm to their partner and/or baby. Furthermore, expectant fathers feared being excluded or invisible and becoming only a spectator during the birth of their baby:

*‘The fact that the professionals draw expectant parents into the childbirth process will strengthen my self-confidence and commitment, which could reduce my worries and anxiety about what is happening … It does not have to be much more than making eye contact and having some questions addressed.’* (Participant 4)

If expectant fathers lacked sufficient professional support, they said they would feel even worse, be disappointed and annoyed, and not know how to behave. Instead, the expectant fathers wished that the midwives would trust and understand their fear, take them seriously and provide professional support to them as well to help them cope with the situation.

All expectant fathers reported that they were well supported by the midwife during labor and birth by their professionalism, approach, and communication skills. They considered the midwives as being professional when they were experienced, skilled, and knowledgeable, had control, continuously checked up on them and found solutions to difficulties they faced. The fathers also felt supported by the midwife’s approach when being calm, present, engaged, involved, friendly, jocular, showing respect and confirmation of the father’s presence. The expectant fathers valued being involved in the care given during birth. Fathers stated that midwives had strong communication skills when midwives explained all of the steps in detail and answered their questions clearly. Fathers felt that they could ask whatever they wanted and said the staff both informed them and listened. When being well supported, fathers felt involved, not forgotten, in control, safe and looked after. When fathers experienced support, they were able to trust and use their advice, rather than having their own solutions. One deficient support aspect was however mentioned, where fathers did not like having shift-changes, and therefore had to meet and discuss their birthing situation with new staff.

Fathers’ childbirth fear was exacerbated by not being able to handle the unknown, unpredictable process of childbirth. The unknown included not knowing what to expect during birth, what kind of situations would evolve, not knowing what to prefer during birth, and how to act, think and react. These feelings led to an experience of being left out, powerlessness, loss of control and doubt if they could manage to be part of the labor and birth. The sense of not being able to do something about the situation made it even more difficult. Because of the unknown process, they found it difficult to prepare for birth:

*‘To a certain extent, it is completely uncertain … so you just have to try to deal with it and try to handle yourselves and your feelings.’* (Participant 4)

On the other hand, expectant fathers suggested that the unknown childbirth also could have positive implications:

*‘To think positively about it instead … that the unknown doesn't have to be bad, it could very well be fantastic.’* (Participant 5)

When the process of labor and birth was uncomplicated and experienced as quick, smooth, and better than expected, the fathers had a positive birth experience. Fathers felt positively if they had no time to reflect on what might go wrong. The unknown labor and birth process did not become scary; instead, spontaneously, the fathers experienced it as positive, exciting, interesting, wonderful, joyful, and fun:

*‘Once we were there in the birthing room and everything was happening, the unknown was no longer creepy; the creepy and dark thoughts never showed up.’* (Participant 5)

Fathers’ FOB also included not knowing how to become a good father. Fear related to their parental role was described as a dreaded delight as they greatly welcomed and valued having a baby but were worried about raising a baby alone, doubted their parental abilities, and felt they would not have the financial resources necessary for raising a child. They mentioned that they would have appreciated if their own father had talked about their experiences of fatherhood. However, positive experiences were also described related to fatherhood:

*‘Just happiness, I love children! A miracle, a kind of exhilaration, a kind of buzz in my stomach and an anticipation.’* (Participant 4)

### Theme 2: Helpful strategies for coping with fear

According to the fathers, an important strategy for dealing with childbirth fear was to share the fear with their partner, other relatives, other men with childbirth fear, professionals and to take part in the present study without receiving any answers or solutions. Expectant fathers could talk individually or in groups about their fear. The opportunity to be offered individual support was valued because the couple could have different personalities, being fearful for different issues and need different kinds of support:

*‘Mothers are not going to be fathers, and fathers are not going to be mothers … it is easier to talk when my partner is not present.’* (Participant 4)

Being given the opportunity to talk about their childbirth fear was considered very important in developing realistic childbirth expectations. Through conversations, they would have the opportunity to put words to their experiences, which could then lead to reconciliation. Fathers stated that talking about their fear led to reflection, confirmation, and preparation for different birth scenarios:

*‘If you talk about the ghost, it will disappear; otherwise, it will just grow bigger inside your head.’* (Participant 4)

Fathers noted that it could help to meet other fathers and share experiences of fatherhood, allowing them to give and receive advice and to understand that everyone has a limited understanding of childbirth. By connecting to others with similar fears, fathers believed their fears could be changed to feeling safer:

*‘It can help to understand that you are not alone in having this experience of absence of control, but it is equal for everyone.’* (Participant 4)

Fathers mentioned that they could receive professional support by talking to midwives, obstetricians, and/or psychotherapists, but that they were not so keen to ask for support:

*‘A man doesn't cry. Men are not very keen at asking for help, but in the end, you can no longer cope.’* (Participant 6)

To talk with a professional with knowledge about family processes was also mentioned as helpful because parenthood also involves the relationship with their partner:

*‘As an expectant father, I need to be better prepared for practical [issues] and for sex life.’* (Participant 4)

Professionals with medical expertise were valued, but fathers noted that female professionals may not always able to understand the transition to fatherhood. One expectant father stated he was not fearful during the remainder of their pregnancy, as he had received valuable support from a psychologist.

Fathers stated other helpful strategies for coping with childbirth fear, including being knowledgeable about pregnancy, labor, birth, breastfeeding, early parenthood, and fatherhood. Fathers thought it was natural to prepare for childbirth and fatherhood. Participants who already were fathers felt more prepared and safer for the upcoming birth than first-time fathers, as they knew more of what to expect during childbirth. However, fathers reported that they would not know if their preparation helped them cope until after childbirth:

*‘It is uncertain how one will react … if it goes down in flames, it does.’* (Participant 4)

Sources of knowledge mentioned were blogs, books, documentaries about fatherhood and dialogs with friends and the midwife. Fathers highly valued prenatal parental education courses and wanted to prepare together with their partner, which helped them feel like a team.

Increased knowledge could give answers to questions, to receive handy tips about what was going to happen during childbirth and how to give their partner sufficient support, like massages and breathing techniques. To gain knowledge was also regarded as a mental preparation for the birth of their baby that led to having a sense of security, being sensitive and paying attention:

*‘You somehow begin to visualize how this very uncertain birth actually can happen and work out.’* (Participant 4)

Another helpful strategy for coping with childbirth fear was to learn techniques regarding how to think differently, to learn a helpful approach toward the fear they experienced, to learn what triggers the fearful response, and to learn techniques so that they did not faint. However, seeking professional support sometimes led to issues beyond their childbirth fear: *‘The conversation with the therapist was interesting; we talked more about my relationship with my parents and my control needs than my childbirth fear.’* (Participant 6)

The approach to accept, to take it as it comes, to be focused on the partner, and the practical details rather than one’s fear, were mentioned both during pregnancy and after the birth of their baby as helpful strategies to cope with fear:

*‘The fears completely disappeared because I focused so much on looking after my partner.’* (Participant 3)

One father had received cognitive behavioral therapy during pregnancy and had experienced:

*‘… a strong desire to escape during the whole birth, but at the same time I wanted to stay because it went well; very contradictory. It was like one long CBT (Cognitive Behavioral Therapy) exercise, to be exposed to things that are difficult to handle.’* (Participant 7)

Another strategy for dealing with childbirth-related fear was to distance themselves from fear by not thinking, talking, or reflecting on the fear:

*‘I have avoided dealing with this. It's taboo. You don't talk about it … men should be strong and manage everything … but it's difficult to escape. Yes, it is better to do something about it.’* (Participant 7)

To gain a sense of control, which helped reduce their fear, fathers reported strategies such as wanting an elective caesarean delivery. By doing this, they felt the birth would have fewer surprises, and they could better prepare for the birth. Fathers further reported not bonding with their baby before knowing if the baby would survive, as a coping strategy. They felt that if they were not connected to their baby, then they would be less affected should something go wrong.

## DISCUSSION

This study, to our knowledge, is the first qualitative study prospectively following expectant fathers with childbirth fear through the baby’s birth. Fathers’ fears mainly revolved around the health and well-being of their partner and their unborn baby. A feeling of not being able to support their partner adequately during birth was common. Fearful fathers felt vulnerable and therefore required receiving professional support, being allowed to take an active role in the birthing process and having an uncomplicated childbirth were seen as ways of reducing their fear of childbirth.

The data analysis explored the overarching theme: ‘Being vulnerable during the transition to fatherhood’. As expectant fathers take a more active role during birth, feelings of anxiety and vulnerability can arise^1,11,19^. FOB experiences might have an impact on fathers’ mental health, for example with a mental preoccupation about their fear or having a sense of increased vigilance^20^. In the current study, fathers went through a transition where childbirth fear was prominent in their everyday life during pregnancy, but less so after the birth of their baby. As previously described, fear of danger in relation to childbirth are explained by fathers as the risk for childbirth complications, labor pain, if an emergency caesarean delivery is required, and death related to the baby and/or their partner^3,11,12^. If fathers are prepared in advance about childbirth risks, especially via receiving professional support, it may reduce their levels of fear and sense of helplessness^21^.

Fathers in the present study were afraid of not being able to provide sufficient support to their partner during the birth of their baby, but at follow-up interviews, most fathers realized that they could and did support their partner. According to a meta-synthesis, fathers were keen to provide physical and emotional support to their partner during labor and birth^2^. A systematic review reveals that fathers were trying to keep strong for their partner and not upset her^20^.

Fathers with fear of birth may need support from midwives to be able to support their partner^11,22^. In this study, when midwives provided opportunities for fathers to talk about their childbirth fear and respond with empathy and care, fathers felt respected. Their fear was reduced however when midwives shared information and gave explanations about the birth process and in potential next steps. Previously, first-time fathers have felt supported by midwives when they spontaneously explained what and why things happened and were given understandable and honest answers to their questions. However, when midwives did not provide answers to their questions, fathers felt irritated and stopped talking to the midwives^23^.

As noted in the current study, fathers did not always know how to be a good father and would have appreciated if their own father had talked about their experiences of parenthood. Fathers felt left alone with few generational or cultural references of support^24^. Similar to previous research^20^, the current study further noted that fathers could have a fear of talking about their childbirth fears due to socially constructed ideas of gender, where they should be strong and look after their partners’ needs, even when doing so negatively affects their own needs^20^.

When fathers receive midwifery support, they feel useful and empowered, which then helps them further to feel safe, relaxed^2^, and able to cope with birth^11^. It was evident that among the fathers in the current study, FOB was related to a lack of support from midwives. Fathers’ level of involvement in the birthing process depended on the quality of the affirmational support provided by the clinical professionals^2,5^. However, during labor and birth, fathers in the present study were empowered when midwives guided and directed them on what they could do for their partner, allowing them to take an active supporting role in the birth. Similar to our results, previous research highlights that when fathers are given the opportunity to develop realistic childbirth expectations and ways to support their partner, then they have less postnatal anxiety^25^. Since fathers were so well supported by midwives during birth, fathers’ fears may have been reduced and allowed them to have a more satisfactory birth experience.

### Methodological considerations

Applying a qualitative longitudinal prospective study design was beneficial for exploring how men interpreted and responded to their transition to fatherhood, and to identify and understand the meaning of change^14^. The findings need to be transferred with caution for several reasons, including: 1) only fathers with self-reported FOB contacted the researchers to participate; 2) only fathers living in the capital of Sweden were included in the current study; and 3) the fathers who had chosen to participate in the study were having singleton, planned births, and were cohabiting. However, we did include both first-time fathers and fathers with previous children, and all fathers who self-reported FOB also completed the FOBS, which further highlighted that they did have a fear of childbirth. A potential confounder did occur, where one father participated in CBT during the pregnancy, which may have influenced his postnatal FOB. However, since all fathers’ FOB dramatically declined in the postnatal period, this particular father’s score may not have been affected by CBT, while his ability to cope during the current childbirth benefitted from his received CBT. More active recruitment strategies such as, if midwives took on more active recruitment by routinely screening expectant fathers for FOB, it may be helpful for future father recruitment and for understanding the prevalence of FOB in fathers. The data collected gave a rich and deep insight into the phenomena under study, and saturation of the findings was achieved. All authors had substantial experience in qualitative research methods, and the first and last authors are clinically licensed midwives with decades of experience.

## CONCLUSIONS

Fathers’ fears were mainly around the health and well-being of their unborn baby and partner, as well as not feeling like they could adequately support their partner during birth. Helpful strategies for coping with fear were suggested by the fathers and included being able to talk about their fears, to learn more about childbirth, to ignore their fears, to receive professional support, to have a partner with an uncomplicated childbirth and to be involved in the birthing process. After the birth of their baby, the thought of another child in the future did not evoke the same strong feelings of FOB. Expectant fathers may benefit from participating in routine fear of birth screenings and receiving individualized brief interventions for their childbirth fear. It is important to support men in their transition into fatherhood, to help reduce their FOB, and to destigmatize masculinity norms.

## Data Availability

The data supporting this research cannot be made available for privacy reasons, and data were collected in Swedish.
